# Correction: Cold-inducible RNA-binding protein (CIRBP) adjusts clock-gene expression and REM-sleep recovery following sleep deprivation

**DOI:** 10.7554/eLife.113484

**Published:** 2026-10-19

**Authors:** Marieke MB Hoekstra, Yann Emmenegger, Jeffrey Hubbard, Paul Franken

**Keywords:** Mouse

Hoekstra MMB, Emmenegger Y, Hubbard J, Franken P. 2019. Cold-inducible RNA-binding protein (CIRBP) adjusts clock-gene expression and REM-sleep recovery following sleep deprivation. *eLife* **8**:e43400. doi: 10.7554/eLife.43400.

Published 25 February 2019

A collaborator brought to our attention an error in equation (3) in the Materials and Methods section “**Experimental protocol and data acquisition**”.

Equation (3) should read:

$$\displaystyle  t(^\circ {\rm C})=\left [\frac{1}{\beta}*{\rm ln}\left [\frac{R_{t}}{R_{25^{\circ } C}}\right ]+\frac{1}{T_{25^{\circ } C}}\right]^{-1}-273.15$$

The original version of equation (3), shown here for reference, read:

$$\displaystyle  t(^\circ {\rm C})=\left [\frac{1}{\beta}*{\rm log}\left [\frac{R_{t}}{R_{25^{\circ } C}}\right ]+\frac{1}{T_{25^{\circ } C}}\right]^{-1}-273.15$$

This error (*log* instead of *ln*) was introduced when preparing the methods section of the manuscript. The correct equation was, however, used in the analysis code that produced the results shown in the paper.

The article has been corrected accordingly.

