# Supplementary material for: Cold-inducible RNA-binding protein (CIRBP) adjusts clock-gene expression and REM-sleep recovery following sleep deprivation
Source: eLife. 2019 Feb 5;8:e43400. doi: 10.7554/eLife.43400 (PMC6379088; doi:10.7554/eLife.43400)
Supplement: Transparent reporting form [file elife-43400-transrepform.docx]

***eLife’s* transparent reporting form**

We encourage authors to provide detailed information *within their submission* to facilitate the interpretation and replication of experiments. Authors can upload supporting documentation to indicate the use of appropriate reporting guidelines for health-related research (see [EQUATOR Network](http://www.equator-network.org/%20)), life science research (see the [BioSharing Information Resource](https://biosharing.org/)), or the [ARRIVE guidelines](http://www.plosbiology.org/article/info:doi/10.1371/journal.pbio.1000412) for reporting work involving animal research. Where applicable, authors should refer to any relevant reporting standards documents in this form.

If you have any questions, please consult our Journal Policies and/or contact us: [editorial@elifesciences.org](mailto:editorial@elifesciences.org).

**Sample-size estimation**

- You should state whether an appropriate sample size was computed when the study was being designed
- You should state the statistical method of sample size computation and any required assumptions
- If no explicit power analysis was used, you should describe how you decided what sample (replicate) size (number) to use

Please outline where this information can be found within the submission (e.g., sections or figure legends), or explain why this information doesn’t apply to your submission:

The sample size determination is partly based on our previous publications in which we quantified the sleep deprivation incurred changes in cortical gene expression using at least 5 mice per experimental group. In keeping with this we included 5 samples per genotype per experimental condition, leading to 30 samples per tissue in total (5 samples x 2 genotypes x 3 conditions). Post-hoc effect size calculation supports this number; the attenuated sleep-deprivation induced changes in cortical *rev-erbα* in *Cirbp* KO mice compared to WT mice at ZT6 has a Cohen’s d effect size of 1.85; a very large effect size.

In our sleep/EEG analyses, many (independent) outcome variables are evaluated each with their own variance, thus requiring varying sample sizes for a desired power of 0.8 and significance level (alpha) of 0.05. For example, in our study the diminished recovery of REM sleep in *Cirbp* KO mice compared to WT mice has a Cohen’s d effect size of 1.3, whereas the accelerated theta peak frequency in *Cirbp* KO mice has a much smaller effect size (Cohen’s d; 0.65). We therefore adhered to our own experience.

**Replicates**

- You should report how often each experiment was performed
- You should include a definition of biological versus technical replication
- The data obtained should be provided and sufficient information should be provided to indicate the number of independent biological and/or technical replicates
- If you encountered any outliers, you should describe how these were handled
- Criteria for exclusion/inclusion of data should be clearly stated
- High-throughput sequence data should be uploaded before submission, with a private link for reviewers provided (these are available from both GEO and ArrayExpress)

Please outline where this information can be found within the submission (e.g., sections or figure legends), or explain why this information doesn’t apply to your submission:

Information concerning replicates can be found in the section ‘statistics’ of Material and Methods. Outliers are discussed in ‘statistics’, ‘analysis based on EEG state’ and ‘analysis of cortical temperature’. Experiments were performed several times to reach the n as discussed in sample size estimation (biological replicates); the experimental batches are indicated in Material and Methods (‘EEG/EMG and thermistor implantation’ and ‘Gene expression in liver and brain’)

**Statistical reporting**

- Statistical analysis methods should be described and justified
- Raw data should be presented in figures whenever informative to do so (typically when N per group is less than 10)
- For each experiment, you should identify the statistical tests used, exact values of N, definitions of center, methods of multiple test correction, and dispersion and precision measures (e.g., mean, median, SD, SEM, confidence intervals; and, for the major substantive results, a measure of effect size (e.g., Pearson's r, Cohen's d)
- Report exact p-values wherever possible alongside the summary statistics and 95% confidence intervals. These should be reported for all key questions and not only when the p-value is less than 0.05.

Please outline where this information can be found within the submission (e.g., sections or figure legends), or explain why this information doesn’t apply to your submission:

The general statistical approach used is explained at the end of the Material & Methods section in the ‘statistics’ section. Statistics performed on the visualized data are mentioned in the figure legend. The n (biological replicates) can be found in the graphs and/or in the text. Statistics on data that is not visualized are mentioned in the main text.

(For large datasets, or papers with a very large number of statistical tests, you may upload a single table file with tests, Ns, etc., with reference to sections in the manuscript.)

**Group allocation**

- Indicate how samples were allocated into experimental groups (in the case of clinical studies, please specify allocation to treatment method); if randomization was used, please also state if restricted randomization was applied
- Indicate if masking was used during group allocation, data collection and/or data analysis

Please outline where this information can be found within the submission (e.g., sections or figure legends), or explain why this information doesn’t apply to your submission:

This information can be found in the section ‘Statistics’ of Material and Methods.

**Additional data files (“source data”)**

- We encourage you to upload relevant additional data files, such as numerical data that are represented as a graph in a figure, or as a summary table
- Where provided, these should be in the most useful format, and they can be uploaded as “Source data” files linked to a main figure or table
- Include model definition files including the full list of parameters used
- Include code used for data analysis (e.g., R, MatLab)
- Avoid stating that data files are “available upon request”

Please indicate the figures or tables for which source data files have been provided:

All figures and ‘child’ figures have their own source data files that contain values of individual observations with which the plots and the statistics can be reproduced. Only data that is based on interval calculations (*e.g.* NREM delta power EEG) is given as mean+/-1SEM, together with the mean timing and +/-1SEM.
